# Behavioral Mechanisms Underlying the Link Between Smoking and Drinking

**Published:** 2000

**Authors:** Hilary J. Little

**Affiliations:** Hilary J. Little, Ph.D., is a professor of psychopharmacology and heads the Drug Dependence Unit, Psychology Department, Durham University, Durham, United Kingdom

**Keywords:** nicotine, neurobehavioral theory of AODU (alcohol or other drug [AOD] use, abuse, and dependence), synergistic drug interaction, impulsive behavior, sensation-seeking behavior, beneficial vs. adverse drug effect, AOD tolerance, AOD sensitivity, stress as an AODC (cause of AOD use, abuse, and dependence), tension reduction theory of AODU

## Abstract

Many people use both alcohol and nicotine (i.e., cigarettes and other tobacco products). The behavioral effects of these two drugs differ, and they do not act on the same target sites in the brain, although they may share, or partly share, certain properties. The initiation of alcohol or nicotine use may be precipitated by similar personality characteristics in the user, such as impulsivity and sensation seeking. Moreover, the mechanisms underlying the development of dependence may be similar for alcohol and nicotine. Thus, certain factors, such as reinforcing drug effects, conditioning processes, automatic behavior, and stress, may influence the development of dependence on both drugs. Other factors, such as tolerance and sensitization to the drugs’ actions and the development of withdrawal symptoms, may also contribute to dependence. This review discusses the actions of the two drugs on certain brain chemical (i.e., neurotransmitter) systems and the extent to which the effects of the two drugs may interact.

The use of drugs^1^ outside clinical medicine is motivated by many factors, including experimentation, peer pressure, self-medication for psychological problems (e.g., anxiety and depression), and dependence. Therefore, the strong association between alcohol consumption and cigarette smoking is also likely to be attributable to multiple factors, including pharmacological actions common to both alcohol and nicotine. This article first explores the reasons underlying initiation of drug use, such as the pharmacological effects of alcohol and nicotine, then reviews the behavioral mechanisms involved in alcohol and nicotine dependence. Whenever possible, these discussions highlight the mechanisms that may account for alcohol and nicotine co-dependence.

## Behavioral Mechanisms Involved in the Initiation of Drinking and Smoking

### Acute Behavioral Effects of Nicotine and Alcohol

**Figure 1:**
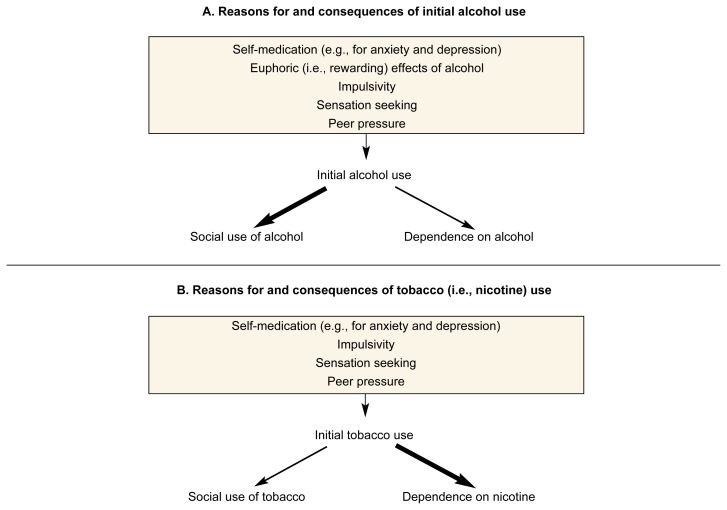
Reasons for and consequences of initial use of alcohol (A) and tobacco (i.e., nicotine) (B). Several factors can motivate a person to drink or smoke during the initial phase (i.e., the first few times) of alcohol or tobacco use. Among drinkers, the majority of people develop a pattern of social drinking; only a minority of drinkers becomes dependent on alcohol. Conversely, the vast majority of smokers become nicotine dependent; only a few smokers maintain a pattern of social use. The thickness of the arrows reflects the proportion of people that develop the drinking and smoking patterns indicated.

The reasons why most people initially^2^ experiment with drugs are related to the drugs’ acute pharmacological effects, such as relief of anxiety or stress and induction of euphoria (see figure 1). These effects result from the drugs’ actions on various brain chemical (i.e., neuro-transmitter) systems in the central nervous system (CNS). These initial target sites in the CNS for alcohol and nicotine differ in many respects. Nicotine interacts with specific “docking molecules” (i.e., protein receptors) on the surface of certain nerve cells (i.e., neurons). In contrast, alcohol produces selective actions on several neurotransmitter systems. (For more information on these actions, see the section “Neuronal Mechanisms Involved in Dependence” on p. 221 of this article).

The acute behavioral effects of alcohol and nicotine and the interactions between these effects have been described in detail (Perkins 1997). As with all drugs that act on the CNS, these effects are crucially determined by the dose that is taken,^3^ as follows:

- At lower doses, nicotine has an alerting effect, resulting in increased attention and improved concentration.
- At higher doses, nicotine has been reported to have a depressant effect on mood and arousal, although this effect is not as pronounced as that of alcohol.
- Low alcohol doses cause such effects as a decrease in the normal social inhibitory control of behavior, loss of motor control, incoordination, and increased reaction times. These behavioral effects of alcohol generally are not shared by nicotine, with the possible exception of a decrease in inhibitory control of behavior that has been suggested by some experimental results (Olausson et al. 1999).
- Higher alcohol doses have effects ranging from intoxication and sedation to general anesthesia.

The increased ability to concentrate after nicotine ingestion is thought to be one reason underlying its use by humans. Studies in humans have also shown that nicotine decreases the sedative properties of alcohol (Perkins 1997), an effect that may promote the combined intake of the two drugs.

The possible presence of a withdrawal state and the influence of such a state on measurements must be considered when assessing the acute effects of alcohol and nicotine. This influence may differ for alcohol and nicotine, because the rate with which people develop dependence on these drugs differs. Thus, dependence on nicotine appears to develop rapidly (i.e., possibly within months). Moreover, high proportions of people who try nicotine become dependent on it. In contrast, alcohol dependence develops more slowly (i.e., normally over several years) and is seen in only a small proportion of people who regularly drink alcohol. Consequently, in studies evaluating the effects of these drugs (e.g., in volunteer populations), investigators must consider the extent to which the subjects are dependent on the drugs. For example, people who regularly smoke cigarettes are likely to be dependent on nicotine, and this dependence may influence the effects of nicotine when the volunteers have been asked to stop smoking, even for a couple of days. This finding is particularly relevant to the estimation of the anxiety-reducing and euphoric effects of nicotine. Conversely, many people who use alcohol regularly do not show symptoms of dependence, and thus abstinence from alcohol for a few days will have a lesser influence on the acute actions of alcohol in volunteers.

### Anxiety-Reducing and Antidepressant Effects of Alcohol and Nicotine

Many people use drugs for self-medication of psychological problems, and both alcohol and nicotine have been suggested to have anxiety-reducing (i.e., anxiolytic) and antidepressant effects. The use of alcohol for the relief of anxiety, particularly in social situations, was described many years ago in the “tension-reduction” hypothesis. Nicotine has also been said to have anxiolytic properties; however, these properties have been observed primarily in nicotine-dependent people, in whom the drug may act by preventing or relieving the anxiety-producing (i.e., anxiogenic) effects caused by nicotine withdrawal.

In animal experiments, researchers have assessed the anxiolytic effects of both alcohol and nicotine using the elevated plus maze test. In that test, rodents are placed in a plus-shaped maze that is elevated above the ground. Of the four arms of the maze, two are open and two are enclosed. The investigators then measure the amount of time the animals spend in the enclosed or open arms of the maze. Because open spaces are a potentially threatening environment for rodents, an increase in the time spent in the open arms after drug administration is thought to reflect an anxiolytic drug action, although this assumption is not entirely uncontroversial (Olausson et al. 1999). Both alcohol and nicotine have positive effects in this test. Furthermore, these effects may be additive—that is, when alcohol and nicotine are used together, the anxiolytic effect is greater than when either drug is used alone (Onaivi et al. 1989). Other studies, however, have suggested that nicotine has an anxiogenic effect.

The occurrence of both clinical depression and dependence on drugs in the same person (i.e., comorbidity) is common, and depression may be a contributing factor in the development of both nicotine dependence and excessive alcohol use. In many cases, however, it is difficult to determine whether pre-existing depression leads to excessive drug use and dependence or whether depression is the result of drug use (Markou et al. 1998). Animal experiments that are thought to predict antidepressant properties of drugs have suggested that both alcohol and nicotine may have such an effect (Ciccocioppo et al. 1999; Semba et al. 1998). The mechanisms underlying these effects, and whether the combination of alcohol and nicotine has a greater effect than either drug alone, however, have not been thoroughly investigated.

### Production of Euphoria and the Rewarding Effects of Alcohol and Nicotine

The term “euphoria” describes the pleasant feeling, or “high,” produced by many drugs and is considered a rewarding effect in humans. In animal experiments, where one cannot directly measure emotions such as euphoria, rewarding effects are defined as those drug actions that promote approach behavior.

People commonly report euphoria after drinking alcohol, although the extent depends on the social setting. This effect is frequently given as an important reason for the social use of alcohol. Smokers also have described euphoria from cigarettes. This effect, however, is not necessarily produced in people who do not use nicotine regularly and may be attributable to removal of the unpleasant experience of nicotine withdrawal rather than an intrinsic euphoric action of nicotine.

In animal experiments, rewarding properties of drugs are tested using an experimental design called conditioned place preference, which measures the extent to which a rodent chooses to be in a location where it has previously experienced the effects of a drug. This experimental design has the advantage that the test measurements are made in the absence of the drug; therefore, such drug effects as motor impairment or sedation do not confound the results. However, the procedure normally requires repeated drug administration to produce the association between the place in which the animal is located and the effects of the drug (i.e., conditioning). Consequently, adaptive changes in brain function may occur during this procedure, and the test results may not reflect initial drug effects.

Both alcohol and nicotine have been shown to cause approach behavior to the paired environment in the conditioned place preference test. For nicotine, a complex relationship exists in these studies between the nicotine dose administered and the effects in the test. For alcohol, conditioned place preference has been reported to occur only after longer periods of conditioning than are required for other psychostimulant drugs (e.g., amphetamine), and differences among species have been found. Thus, it can be difficult to produce such an effect in rats, whereas an effect can be seen in mice after relatively short conditioning periods. There has been limited investigation of the extent to which the combination of alcohol and nicotine shows additive effects in this test.

Relatively strong evidence indicates that the acute euphoric actions of alcohol contribute to its use in humans, although evidence of this same effect from nicotine is weaker. It will be important for researchers to establish whether using alcohol and nicotine in combination increases the euphoric, and therefore rewarding, actions of these drugs or whether concomitant alcohol drinking may perhaps result in a euphoric effect of nicotine in humans that is not seen when nicotine is used alone.

There has, however, been little experimental investigation of such interactions in humans. In one study, participants reported greater subjective enjoyment of cigarettes during alcohol consumption, although this finding was not reflected in their subsequent preferences for cigarettes that were similar (i.e., of the same color) to those they had smoked while under the influence of alcohol (Glautier et al. 1996). Experimental investigation of nicotine’s euphoric effects in humans is complicated both by the need to remove the potentially confounding effects of nicotine withdrawal if tests are conducted on smokers and by the ethical considerations (i.e., the strong potential for inducing dependence) of examining the behavioral effects of nicotine in nonsmokers.

Another experimental design thought to be related to rewarding properties of drugs is intracranial self-stimulation. Animals will voluntarily press a lever to receive an electrical stimulation from electrodes implanted in certain brain areas, particularly the hypothalamus and the ventral tegmental area. Conversely, the animals show no apparent interest or even actively avoid stimulation of many other areas of the CNS. Drugs, including both alcohol and nicotine, lower the thresholds for such self-stimulation (i.e., the threshold at which such responding is initiated). Schaefer and Michael (1992) found additive effects of alcohol and nicotine on self-stimulation of the lateral hypothalamus, but only with one dose combination of the two drugs.

### Aversive Effects of Alcohol and Nicotine

Both alcohol and nicotine frequently have unpleasant effects that are aversive (e.g., nausea) and which depend on the dose of and previous experience with the drug. For both drugs, the aversive properties, at least in part, result from irritation of the stomach. Some of alcohol’s unpleasant effects are caused by acetaldehyde, the first breakdown product (i.e., metabolite) of alcohol. However, these two factors may not explain all the aversive effects of nicotine and alcohol. The development of tolerance to these aversive effects may be a contributing factor in the continued use of both alcohol and nicotine. Laboratory evidence suggests that administration of either alcohol or nicotine and development of tolerance to its aversive effects can also decrease the conditioned taste aversion to the other drug (Kunin et al. 1999). If confirmed in humans, this phenomenon, which is called cross-tolerance, may have important implications for the combined use of nicotine and alcohol in humans.

### Impulsivity and Sensation Seeking

Two additional factors involved in the initiation of drug use are impulsivity and sensation seeking. The term “impulsivity” describes an individual’s tendency to make rapid behavioral changes regardless of detrimental consequences or the loss of a later reward of greater magnitude (e.g., taking a drug despite knowing the potential adverse effects on health or wealth). In laboratory animals, researchers can measure the degree of impulsivity by comparing whether rodents preferentially press a lever to receive a larger, delayed food reward or a smaller, rapidly available reward. Another strategy to assess impulsivity (called differential reinforcement of low rate responding [DLR]) involves tests in which low rates of responding are selectively rewarded. Researchers have identified several different forms of impulsivity, at least some of which involve reduced transmission of nerve signals involving the neurotransmitter 5-HT (also known as serotonin).

The association between impulsivity and alcohol and nicotine use has two important aspects. The first aspect is the extent to which an individual’s innate impulsive behavior results in increased alcohol or nicotine use, often despite knowledge of the adverse consequences. Dependence on both alcohol and on nicotine has been found to be associated with high levels of impulsivity (Poulos et al. 1998; Mitchell 1999). This impulsivity is thought to contribute to the person’s initial use of the drugs and possibly to the development of dependence. The second aspect is the propensity of the drugs themselves to increase impulsivity. There is little direct evidence that nicotine affects impulsivity. Alcohol consumption has resulted in increases in measurements of impulsivity (Poulos et al. 1995). An increase in impulsive behavior caused by alcohol consumption would be expected to increase cigarette smoking during drinking.

Sensation seeking is defined as an individual’s desire for novel or arousing experiences. Studies in humans have found strong correlations between high ratings of sensation seeking and experimental use of drugs, including alcohol and nicotine (Zuckerman 1994). It is as yet unclear whether sensation seeking also promotes the concurrent use of alcohol and nicotine, although this appears likely. Similarly, researchers have not yet identified the neurochemical basis of the association between sensation seeking and drug use. It has been suggested that brain signaling pathways involving the neurotransmitters dopamine and serotonin may play a role. In addition, the effects of alcohol and nicotine on stress hormones (which are discussed in the section “The Influence of Stress” on p. 222 of this article) may be involved both in the initial use of these drugs in an experimental way—for example, by adolescents—and in the continued drug use during dependence.

## Mechanisms of Dependence

The American Psychiatric Association (1994) has defined specific criteria for a diagnosis of dependence in its *Diagnostic and Statistical Manual of Mental Disorders, Fourth Edition* (DSM–IV). These criteria include the development of tolerance and the appearance of withdrawal symptoms when drug consumption is discontinued after prolonged heavy use. Other criteria describe the pattern of compulsive use that is the predominant characteristic of dependence on drugs, together with its long-term nature. Thus, addicts can revert to drug taking and frequently experience all the symptoms of dependence after weeks, months, or even years of abstinence. Such relapse occurs with both alcohol and nicotine, and it is important from a clinical perspective to know whether the continued use of one drug affects the patient’s success in abstaining from the other drug. For example, alcoholics attempting to abstain from alcohol most commonly continue smoking. Accordingly, clinicians need to know whether this continued smoking has adverse effects on abstinence from alcohol, as might be the case if there are common mechanisms of dependence, or whether the difficulties of giving up smoking in addition to alcohol may make life harder and create a more stressful situation for the alcoholic. No extensive evidence regarding these issues exists to date; however, several studies have indicated that giving up smoking as well as drinking does not reduce abstinence rates from alcohol and that it may actually be beneficial to abstain from both alcohol and nicotine simultaneously (Breslau et al. 1996).

Researchers must learn more about the mechanisms underlying the dependence on alcohol and nicotine to maximize the success of treatment for both addictions. Some of those mechanisms are described in the following sections.

### Tolerance and Sensitization After Prolonged Use

**Figure 2:**
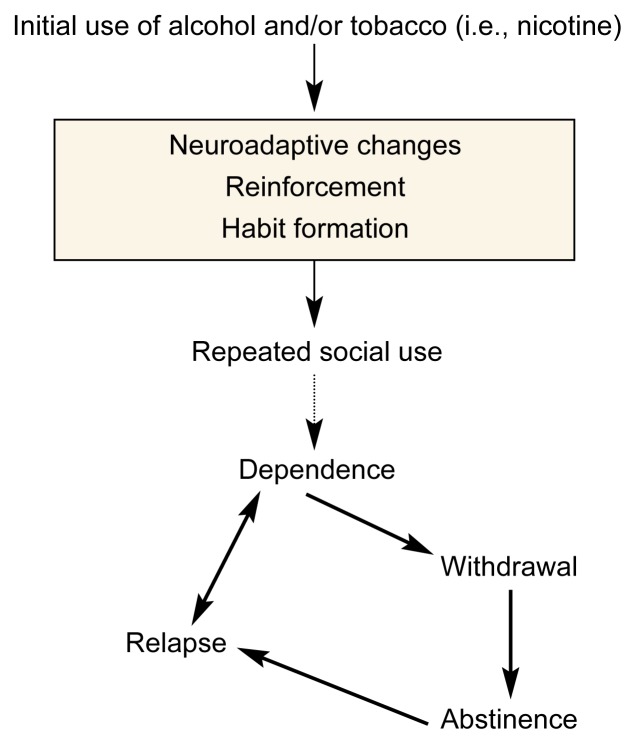
Consequences of continued alcohol and tobacco (i.e., nicotine) use. During the initial use of alcohol and tobacco, changes in the activity of nerve cells (i.e., neuroadaptive changes) occur, resulting in reinforcement and habit formation. These changes lead to repeated social use, which may result in dependence in some people. In those people, sudden abstinence from alcohol or nicotine leads to withdrawal symptoms, which cease during prolonged abstinence. However, even after prolonged abstinence, relapse may occur.

The effects of alcohol and nicotine on brain neurons and on behavior change when these drugs are taken repeatedly or over long periods (see figure 2). As previously described, the rate of development of dependence on nicotine is faster than that on alcohol, and the neuroadaptive changes need to be evaluated in this context. To what extent tolerance contributes to the development of dependence on alcohol and nicotine is a matter of debate. Tolerance to the unpleasant effects of a drug, which is thought to develop with both alcohol and nicotine, is likely to increase drug use, as described earlier in this article. Tolerance to the pleasant effects of a drug, which develops at least with alcohol, also may result in the drug being taken in larger doses in order to achieve the same effect. Furthermore, cross-tolerance between the behavioral effects of alcohol and nicotine has been seen in experimental studies—that is, animals that develop tolerance to the effects of one drug may also become tolerant to the effects of the other drug (e.g., Collins et al. 1993)—but the situation is complex and depends on the measurement and the strain of rodent.

In recent years much attention has been paid to sensitization, which is an increase in the effects of drugs of dependence, including alcohol and nicotine, seen after repeated intake. This sensitization is a long-lasting effect and therefore is a prime candidate for the prolonged nature of dependence. There is considerable uncertainty, however, as to whether sensitization occurs in humans. The “incentive-sensitization” theory of Robinson and Berridge (1993) suggested that the “incentive salience” (i.e., the importance to an individual) of drugs of dependence and of stimuli associated with the drugs increases as these drugs are taken repeatedly and therefore acquire strong motivating properties and control over behavior.

Animal studies have focused primarily on sensitization to the effects of drugs on locomotor activity. Sensitization to the locomotor stimulant actions of both nicotine and alcohol occurs in experimental animals, although with considerable strain differences in the case of alcohol. Researchers also have reported cross-sensitization between drugs, including those from different pharmacological classifications, such as psychostimulants and opiates. Chronic alcohol consumption was found to increase the locomotor activity following repeated injections of nicotine (Watson and Little 1999). If a similar situation existed in humans (i.e., if prior alcohol consumption could enhance the effects of nicotine), this would have important implications for the combined use of the two drugs.

### Withdrawal Symptoms

The appearance of a withdrawal syndrome after cessation of long-term drug consumption is an important criterion in the diagnosis of dependence and in the definition of a drug’s dependence liability. The importance of the withdrawal syndrome in the genesis of dependence, however, is now uncertain. The acute manifestations of withdrawal differ for nicotine and alcohol. Tremor, convulsion, and hallucinations are evident following cessation of long-term alcohol consumption, whereas nicotine withdrawal is associated predominantly with irritability, sleep disturbances, and hunger. There is some similarity, however, in the occurrence of anxiety and unhappiness (i.e., dysphoria) in both syndromes. It is still unknown whether simultaneous withdrawal from alcohol and nicotine results in a more severe syndrome than withdrawal from each drug separately. Some laboratory evidence suggests, however, that this may indeed be the case (e.g., Onaivi et al. 1989).

The symptoms described above are the subjective feelings seen immediately after withdrawal from chronic intake of the drugs and last a relatively short time after cessation of drug use. The compulsive drug taking, however, lasts far longer, which has led many researchers to discount withdrawal as an important factor in dependence, although addicts often report taking drugs to remove the subjectively unpleasant experience of withdrawal as a major reason to continue drug taking. In addition, treatment of the acute withdrawal symptoms does not prevent dependence on drugs. The importance of withdrawal signs, however, depends on the duration of withdrawal that is considered, and recently attention has been turning to the investigation of behavioral and neurochemical changes that occur or persist at longer times into the abstinence period. Conditioned withdrawal (which is discussed in the section “Conditioning and Automatic Behavior” on p. 222 of this article) can also occur after considerable duration of abstinence.

### The Reinforcing Effects of Alcohol and Nicotine

Reinforcing effects are those actions of drugs that increase the relationship between a stimulus (e.g., presentation of an alcoholic beverage) and a response (e.g., drinking). In behavioral terms, a reinforcing effect of a drug will promote the behavior that preceded the stimulus. These reinforcing properties of drugs are often confused with, and equated with, the rewarding effects of drugs, which have been discussed earlier. However, the two properties are not identical, and the distinction has been elegantly discussed by White (1989). “Reward” involves feelings of pleasure and therefore describes the hedonic or euphoric effects of drugs, whereas “reinforcement” refers to the relationship between the behavior and the stimulus (in this instance, the drug). Many drugs that cause dependence have both reinforcing and rewarding effects, but the reinforcing actions need not involve pleasant or euphoric effects, and some reinforcing stimuli are actually aversive. Negative reinforcement occurs when the response involves avoidance of the stimulus and is used to describe the situation when a drug is taken to remove the unpleasant effects of drug withdrawal. Reinforcement has been equated with memory and learning; however, whereas nicotine can improve memory and learning, alcohol, over the dose range normally consumed by humans, does not have this action and at high doses is amnesic (i.e., causes memory impairment).

In animal experiments, reinforcing effects are measured primarily through a test design called operant self-administration. Laboratory animals learn that performance of a motor task (e.g., pressing a lever) results in the administration of a small dose of a drug (e.g., an intravenous injection of nicotine or presentation of a dipper containing alcohol). The experiments measure the number of times the animal will perform the motor task to obtain small amounts of the drug. These motor tasks frequently involve complex schedules of task performance and drug presentation. The important aspect is that the lever pressing provides a measure of the motivation to obtain the drug. For example, an animal will perform large numbers of lever presses for one drug dose only if the animal is strongly motivated to obtain the drug. Both alcohol and nicotine are voluntarily self-administered by laboratory animals. For studies assessing alcohol self-administration, however, the situation is complicated by the fact that many animals find the taste of alcohol aversive. Consequently, operant self-administration is normally induced by training the animals to respond for sweet solutions containing alcohol, analogous to the situation in many humans who are introduced to alcohol in the form of sweetened and flavored drinks.

Operant self-administration studies on the interactions between alcohol and nicotine have not given consistent results. Thus, nicotine injections decreased self-administration of alcohol (Nadal and Samson 1999; Nadal et al. 1998). However, an agent that counteracted nicotine’s effects (i.e., a nicotine antagonist) called mecamylamine had a similar effect on alcohol self-administration (Nadal et al. 1998). Chronic infusion of certain doses of nicotine, however, increased operant self-administration of alcohol (Clark et al. 1998).

One aspect that should be taken into consideration here is that a large proportion of such research has concentrated on psychostimulant drugs, amphetamine, and cocaine. Animals will press levers at high rates to obtain intravenous injections of these drugs, and this is reflected in the experimental designs. Alcohol and nicotine have been said to possess weaker reinforcing properties, because they do not support such high rates of responding. The human evidence indicates clearly the high-dependence liability of both nicotine and alcohol, however, and it is possible that the apparently weaker test effects of alcohol and nicotine reflect the fact that the experimental designs are not optimal for these drugs. Stolerman and Jarvis (1995) have pointed out that research on the reinforcing actions of nicotine was many years behind that on other drugs, and it appears that despite improvements, this is still the case.

### Neuronal Mechanisms Involved in Dependence

**Figure 3:**
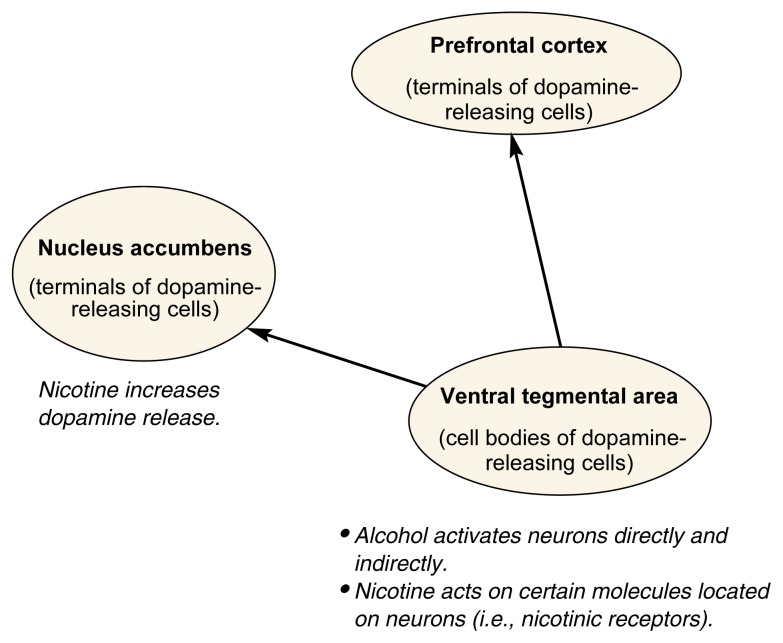
Effects of alcohol and nicotine on the mesolimbic dopamine system. Dopamine is a brain chemical believed to play an important role in addiction. The mesolimbic dopamine system includes three brain regions: the ventral tegmental area, nucleus accumbens, and prefrontal cortex. The ventral tegmental area contains dopamine-releasing nerve cells (i.e., neurons). The extensions of those neurons lead into the nucleus accumbens and prefrontal cortex, where they release dopamine to activate other neurons. The actions of alcohol and nicotine in these brain areas are shown in italic.

To what extent do we understand the underlying neuronal changes responsible for the reinforcing and rewarding effects of alcohol and nicotine and their capacity to produce dependence? One brain system frequently implicated in the reinforcing effects of drugs is the mesolimbic dopamine system, which includes the ventral tegmental area, the nucleus accumbens, and the prefrontal cortex (see figure 3). Substantial evidence indicates that this system plays an important role in the motivation of animals (including humans) to obtain natural rewards, such as food or sex, although not in the consummatory behavior subsequent to gaining such rewards. Although the initial target sites for alcohol and nicotine differ, these drugs both increase the activity of mesolimbic dopaminergic neurons, albeit through different mechanisms. Neurochemical dopamine depletion in the nucleus accumbens decreases operant self-administration of nicotine by rodents (Stolerman and Shoaib 1991) and the acquisition of self-administration of alcohol. However, both dopamine antagonists and dopamine agonists (i.e., agents that act like dopamine) have been reported to decrease responding for alcohol. The role of mesolimbic dopaminergic transmission in the reinforcing effects of alcohol is therefore somewhat uncertain. In addition, the dopamine antagonist haloperidol did not prevent the effect of alcohol in conditioned place preference (Cunningham et al. 1992).

Recent studies have suggested that the mesolimbic dopamine system is more concerned with the importance of environmental stimuli and the choice of behavioral responses to these stimuli. Dopaminergic neurons respond more strongly to novel stimuli than to predictable stimuli (Schultz 1997). In the case of drug dependence, the drugs may produce similar effects on dopamine as does an important stimulus, and thus the drugs themselves become the focus of attention. Researchers do not yet understand, however, how those changes in dopamine transmission take place during the development of drug dependence. It is also worth considering whether rodents that self-administer alcohol are actually dependent on the drug. For example, the animals do not usually show overt signs of behavioral changes or drug seeking when the drug is not available, although they may respond to cues that they have previously associated with the drug.

Some studies have investigated the role of the mesolimbic dopamine system in the interaction between alcohol and nicotine. Blomqvist and colleagues (1997) found that the nicotine antagonist mecamylamine, when injected either into peripheral parts of the body or directly into the ventral tegmental area, could prevent alcohol’s effects on the mesolimbic dopamine system. Prior nicotine administration increased the effects of alcohol on that system (Blomqvist et al. 1993). These findings suggest that alcohol’s actions on the mesolimbic dopamine system involve the activation of nicotine receptors. How these effects relate to alcohol consumption is uncertain, however; as described previously, the effects of nicotine and nicotinic antagonists on operant self-administration of alcohol by rodents are complex.

Another group of brain chemicals thought to be intimately involved in mechanisms of reward and possibly of reinforcement are the endogenous opiates (e.g., enkephalins). Alcohol consumption in both laboratory rodents and alcoholics is decreased by administration of an opiate antagonist, such as the medication naltrexone, which has some efficacy in the treatment of alcoholics (Volpicelli et al. 1995). There appears to be some interaction between endogenous opiates and the mesolimbic dopaminergic system, because the alcohol-induced release of dopamine in the nucleus accumbens is blocked by opiate antagonists (Benjamin et al. 1993). The use of opiate antagonists in nicotine dependence has not yet proved beneficial, although experimental interactions have been reported and this line of approach is being pursued (Pomerleau 1998).

Withdrawal from chronic intake of alcohol or nicotine decreases the release of dopamine in the nucleus accumbens. It has been suggested that such decreased mesolimbic activity causes a lack of ability to experience pleasure (i.e., anhedonia) and that this effect is involved in drug dependence. However, a simple decrease in mesolimbic activity would be expected to lower the ability of drugs to produce rewarding effects. In contrast, recent evidence indicates that changes in the mesolimbic system continue beyond the acute phase of withdrawal, at least in the case of alcohol (Diana et al. 1996; Bailey et al. 1998). It is possible that a decrease in baseline activity of this system could occur in conjunction with increased activation in response to drugs; such an alteration could be involved in relapse to drug taking.

### Conditioning and Automatic Behavior

Conditioning occurs when a stimulus or cue (e.g., food) that results in a specific effect (e.g., salivation) is repeatedly paired with a previously unrelated stimulus (e.g., a certain sound). After a while, the unrelated stimulus becomes a conditioned stimulus that can evoke the same effect, even in the absence of the original stimulus. Conditioning is involved in several ways in dependence on both alcohol and nicotine. Environmental cues present during drug taking (e.g., the sight or smell of a bar) become associated with the drug effect (e.g., physiological responses to alcohol and nicotine), and conditioned responses are then seen in the absence of the drug. Such responses can be either in the opposite direction to the drug’s effects, causing tolerance, or in the same direction, mimicking drug action. Drug-associated cues therefore acquire the ability to control behavior, and this effect has been suggested to be involved in relapse drug taking.

Conditioning factors that contribute to dependence on one drug may increase dependence on another drug, and it is possible that conditioning factors are involved in the cross-tolerance reported between some actions of alcohol and nicotine. Thus, an increase in locomotor activity in an environment in which mice had previously experienced nicotine was seen only in animals that had previously consumed alcohol chronically, suggesting that prior chronic alcohol intake may increase conditioned responses to nicotine (Watson and Little 1999).

Conditioned withdrawal—that is, the precipitation of withdrawal symptoms by environmental cues previously associated with the drug—has been reported for alcohol and for nicotine. This response can be produced at long intervals after cessation of drug use and therefore is likely to contribute to relapse drug use during abstinence.

With repeated practice, many everyday processes (e.g., getting dressed or driving a car) become so automatic that a person can carry them out rapidly, without conscious awareness or involvement of cognitive processes. Such processes form a stereotypic series of actions that is difficult to halt. It has been suggested that automatic processes contribute to the dependence process (Tiffany 1990). According to this theory, repeated association of drug effects with drug-taking behavior causes incremental strengthening of the stimulus-response relationship, resulting in drug taking becoming an automatic process. Such processes may contribute particularly to nicotine dependence, because cigarette smoking involves stereotyped, ritualistic behavioral patterns (e.g., taking the cigarette out of the package and lighting it). It is difficult, however, to reconcile this theory with the occurrence of relapse drinking and smoking that continues despite the knowledge of serious health consequences and the adverse reactions on the part of family and friends, or in situations in which the drug is not easily available. In such scenarios, a person would become much more conscious of his or her behavior (e.g., to obtain a drug), and thus the behavior would no longer be automatic. Binge drinking by alcoholics also does not fit a pattern of automatic behavior. The theory also posits that during co-dependence of alcohol and nicotine, alcohol may act as a triggering cue for the automatic behavior leading to nicotine use as well as interfere with nonautomatic processing aimed at the avoidance of smoking (Tiffany 1990).

### The Influence of Stress

One factor common to the use of nicotine and alcohol is the influence of stress. Traumatic life events and chronic stressful experiences are associated with the development of both alcohol and nicotine dependence. Stress has also been reported to precipitate relapse drug taking (Piazza and Le Moal 1998) and may affect conditioning. Stressful experiences result in the release of stress hormones called glucocorticoids from the adrenal glands, which are located on top of the kidneys. The primary glucocorticoid in humans is cortisol; the corresponding hormone in rodents is corticosterone. Laboratory studies have shown that corticosterone increases alcohol consumption by rodents. Furthermore, stress has been shown to reinstate operant responding for alcohol and nicotine after such behavior had been extinguished by cessation of the drug presentation (Le et al. 1998; Buczek et al. 1999). Both alcohol and nicotine increase release of glucocorticoid hormones, and this effect may contribute to their use. Corticosterone has been shown to be self-administered by rodents and therefore may itself have reinforcing properties (Piazza et al. 1993). The effects of glucocorticoid hormones on the CNS also may be involved in sensation seeking, a currently speculative but interesting line of investigation.

Long-term changes in the control of stress hormone release have been demonstrated after stressful experiences, and these changes may also influence drug-taking behavior and the development of dependence. Stress has been found experimentally to cause sensitization to drugs and may thus increase their rewarding and/or reinforcing actions and incentive salience. It is conceivable that changes in the control of stress hormone release caused by alcohol or nicotine may be involved in co-dependence on both drugs, but more information is needed before such a link can be validated. Prolonged excess alcohol consumption has been reported to affect hormonal responses to stress and to nicotine.

## Conclusions

Many factors that contribute to the initial use of both alcohol and nicotine differ for the two drugs, but some factors may be synergistic. The prolonged nature of dependence on both drugs suggests that long-term changes occur in neuronal activity and that these may be common to both drugs. Considerably more information is needed, however, about the behavioral and neurochemical interactions between the two drugs—in particular, their effects on impulsivity and sensation seeking and in relieving anxiety and depression, as well as their rewarding and reinforcing effects—before co-dependence can be fully understood.

## Footnotes

1 The term “drug” refers to both alcohol and other licit and illicit drugs, including tobacco (i.e., nicotine).

2 In this article, the term “initial use” refers to the first few times a person or animal uses a drug, not just the first time.

3 Because most of these effects were observed in animal studies, one cannot convert measures, such as “high” or “low” alcohol or nicotine doses, into easily quantifiable units, such as drinks or cigarettes smoked.
